# Quantitative Proteomic Analysis Reveals That Central Metabolic Pathways Influence Norfloxacin Susceptibility in *Vibrio alginolyticus*

**DOI:** 10.3390/life16101607

**Published:** 2026-09-25

**Authors:** Chenghao Shen, Yanchang Wu, Jiaqi Tang, Xiaopei Cai, Ying Zhou, Yankai Liu, Ramanathan Srinivasan, Dianqi Zhang, Xiangmin Lin, Ling Lin

**Affiliations:** 1 School of Life Sciences, Fujian Agriculture and Forestry University, Fuzhou 350002, China; 22305025003@fafu.edu.cn (C.S.); 505243075@fafu.edu.cn (Y.W.); 17281182177@163.com (J.T.); 12505014019@fafu.edu.cn (X.C.); 52505043021@fafu.edu.cn (Y.Z.); 13807459688@163.com (Y.L.); 2 Fujian Provincial Key Laboratory of Agroecological Processing and Safety Monitoring, Fuzhou 350002, China; 3 Key Laboratory of Crop Ecology and Molecular Physiology, Fujian Agriculture and Forestry University, Fujian Province University, Fuzhou 350002, China; 4 School of Applied Bioscience, Food and Agritech, Rathinam Global Deemed to be University, Coimbatore 641021, Tamil Nadu, India; srinivasan.bio@rathinam.in; 5 Yunxiao Profitable Feng Aquatic Technology Co., Ltd., Zhangzhou 363300, China; 6 Key Laboratory of Marine Biotechnology of Fujian Province, Institute of Oceanology, Fujian Agriculture and Forestry University, Fuzhou 350002, China

**Keywords:** *V. alginolyticus*, norfloxacin susceptibility, quantitative proteomics, metabolic remodeling, ROS accumulation, reduced glutathione (GSH) levels

## Abstract

Reduced antibiotic susceptibility often involves reversible metabolic reprogramming. Here, data-independent acquisition (DIA)-based quantitative proteomics was employed to profile the response of *Vibrio alginolyticus (V. alginolyticus)* to norfloxacin (NOR) exposure, revealing a global metabolic downshift marked by prominent reductions in central carbon, energy, and amino acid pathways. High-throughput phenotypic screening identified 11 exogenous metabolites, including glycine (Gly) and glutamine (Gln), that significantly potentiated NOR-mediated growth inhibition. Mechanistically, physiological assays demonstrated that glycine supplementation perturbed cellular redox balance, accompanied by elevated reactive oxygen species (ROS) levels and a reduction in the intracellular reduced glutathione (GSH) pool under NOR challenge. Furthermore, mRNA expression analysis (qPCR) revealed that while NOR restricted transcripts of the downstream anaplerotic Gln-to-2-oxoglutarate axis, including the 2-oxoglutarate dehydrogenase complex and NAD^+^-dependent glutamate dehydrogenase, exogenous Gln supplementation effectively alleviated this transcriptional suppression. Collectively, these findings demonstrate that targeted metabolite supplementation overrides defensive low-flux states, forcing metabolic and transcriptional reactivation that intensifies antibiotic efficacy via ROS-mediated oxidative stress and impairment of antioxidant defense.

## 1. Introduction

The global aquaculture industry has expanded rapidly in recent decades, accompanied by escalating disease outbreaks caused by opportunistic pathogens. In 2024, global fisheries and aquaculture production reached a record 223.2 million tons, valued at approximately USD 1.159 trillion [1]. Among the common pathogenic Vibrio species in aquaculture, including *Vibrio alginolyticus*, *V. parahaemolyticus,* and *V. harveyi*, *V. alginolyticus* is a halophilic Gram-negative bacterium of major concern. It causes severe diseases such as hemorrhagic septicemia [2], ulcerative lesions [2], and hepatopancreatic lesions/pathology [3], leading to mass mortality in species such as *Litopenaeus vannamei* (whiteleg shrimp) and *Larimichthys crocea* (large yellow croaker) [4]. To control such bacterial infections, antibiotics including quinolones, tetracyclines, and sulfonamides are widely used. Norfloxacin (NOR), a fluoroquinolone antibiotic, inhibits DNA replication by targeting DNA gyrase and topoisomerase IV, resulting in bactericidal DNA damage [5,6]. NOR has been used in aquaculture settings, where growing reports of resistance highlight the need to better understand bacterial responses to this antibiotic [7]. In addition to genetic resistance, bacteria can survive antibiotic treatment through reversible physiological adaptations, which can include metabolic slowdown and stress responses that reduce antibiotic susceptibility [8]. In *V. alginolyticus*, emerging evidence suggests that changes in the abundance of central metabolic proteins correlate with reduced antibiotic susceptibility and transiently reduced killing under drug exposure [8]. For instance, the downregulation of central carbon and energy metabolism has been linked to acquired fluoroquinolone resistance, potentially reducing the proton motive force (PMF) and limiting drug uptake [9]. Central carbon metabolism, primarily comprising the Embden-Meyerhof-Parnas (EMP) pathway, the pentose phosphate pathway (PPP), and the tricarboxylic acid (TCA) cycle, forms the fundamental bioenergetic and biosynthetic engine of bacterial survival. Beyond driving ATP generation via substrate-level and oxidative phosphorylation, these interconnected metabolic highways function as critical regulatory hubs that dictate cellular redox homeostasis through the production of primary reducing equivalents, including NADH and NADPH [8,9]. Furthermore, oxidative-stress responses can modulate antibiotic outcomes. For example, gasotransmitters such as hydrogen sulfide (H_2_S) and nitric oxide (NO) can alter antioxidant enzymes, including superoxide dismutase (SOD) and catalase (CAT), thereby influencing reactive oxygen species (ROS) accumulation during treatment [10,11]. Despite these insights, the global proteomic response of *V. alginolyticus* to NOR remains poorly characterized. Here, a data-independent acquisition (DIA)-based quantitative proteomics strategy was employed to profile *Vibrio alginolyticus* in the presence and absence of NOR, identifying coordinated reductions within central carbon, energy, and amino acid pathways. Combined with high-throughput phenotypic screening of exogenous metabolic adjuvants, independent physiological and transcriptional validation workflows were integrated to evaluate node-specific susceptibility mechanisms. Physiological assays established that glycine co-treatment subverts redox homeostasis via synergistic ROS generation and severe GSH depletion, while qPCR verification confirmed that glutamine administration successfully overrides the antibiotic-induced transcriptional suppression of the downstream anaplerotic 2-oxoglutarate dehydrogenase axis. Collectively, this study establishes a comprehensive framework showing how targeted metabolite inputs override defensive low-flux bacterial postures, forcing a metabolic and transcriptional reactivation that intensifies antibiotic efficacy via ROS-mediated oxidative stress and impairment of antioxidant defenses.

## 2. Materials and Methods

### 2.1. Bacteria and Growth Conditions

The bacterial strain *V. alginolyticus* NBRC 15630 was preserved in our laboratory. The bacterial cells were cultured in standard Luria–Bertani (LB) broth medium and maintained at 30 °C. Norfloxacin was prepared as a stock solution, sterilized using a 0.22 μm syringe filter, and stored at −20 °C for subsequent use. A single colony was inoculated into 5 mL of LB medium and incubated at 30 °C with shaking at 200 rpm for 16 h. Subsequently, the cultures were inoculated at a 1:100 ratio into 100 mL of fresh LB broth without NOR (untreated control group) or supplemented with 0.064 μg/mL NOR (experimental group) and incubated at 30 °C with shaking at 200 rpm. Both groups were harvested simultaneously at 4 ± 0.5 h post-inoculation, at which point the untreated control group had reached the mid-exponential phase (OD600 ≈ 1.0). Cells were harvested, washed three times with phosphate-buffered saline (PBS, pH 7.4), and lysed by sonication on ice for 30 min (30% power, 9 s ultrasound pulses with 6 s intervals). After sonication, the samples were centrifuged at 8000 rpm for 20 min at 4 °C to collect the supernatant. Protein concentrations were quantified using the Bradford assay [12]. In accordance with standard broth microdilution guidelines, the minimum inhibitory concentration (MIC) of norfloxacin against *V. alginolyticus* was determined to be 0.128 µg/mL Supplementary File S2 (Figure S3). Therefore, an experimental working concentration of 0.064 µg/mL (1/2 × MIC) was selected to establish a standardized sub-inhibitory model.

### 2.2. Trypsin Digestion

Protein samples (50 μg) were extracted from each group, followed by reduction with 10 mM dithiothreitol (DDT) and alkylation with 50 mM iodoacetamide, as previously described [13]. Subsequently, the enzymatic hydrolysis of the protein samples was carried out using the filter-aided sample preparation (FASP) method. The desalting process was performed with a C18 desalting column, as previously reported [14].

### 2.3. DIA-Based LC-MS/MS Analysis

A total of 100 μg of peptides from each sample were pooled, and the fractions were loaded onto an analytical column using an EASY-nano liquid chromatography (nano-LC) system (Thermo Fisher Scientific, Waltham, MA, USA) at a flow rate of 600 nL/min. For mass spectrometry analysis, MS1 scans were acquired over m/z 350–1400 at a resolution of 60,000. The top 20 precursor ions were selected for MS2 analysis via higher-energy collisional dissociation (HCD) fragmentation at a normalized collision energy of 28, with fragment ions detected at a resolution of 15,000. Data-independent acquisition (DIA) was performed under the same LC-MS conditions as described above. The DIA full scan was acquired at a resolution of 60,000 over m/z 350–1400. DIA scan parameters were set to a resolution of 30,000, with an automatic gain control (AGC) target of 3 × 106 and a maximum injection time set to auto.

Qualitative and quantitative analysis of DIA datasets was performed using Spectronaut 20 (Biognosys AG) across three biological replicates per group, with cross-run normalization executed using precursor-based median local normalization. DDA (Data-Dependent Acquisition) raw files were searched against the *V. alginolyticus* protein sequence database (comprising 4661 UniProt entries) to construct a spectral library. The search parameters were configured according to the BGS factory defaults. Both the peptide-spectrum match (PSM) false discovery rate (FDR) and the protein-level FDR were controlled at 1%. For spectral library construction, the top 3–6 fragment ions per peptide were selected, and each peptide was required to contain at least three fragment ions. All results were filtered using a q-value threshold of 0.01, which corresponds to an FDR of 1%. *p*-value estimation was conducted using the kernel density estimator method. The mass spectrometry proteomics data have been deposited in the ProteomeXchange Consortium (https://proteomecentral.proteomexchange.org) via the iProX partner repository [15,16] with the dataset identifier PXD072925.

### 2.4. Bioinformatics Analysis

Based on the MS results, the online STRING database (https://string-db.org) was used to perform Gene Ontology (GO) and Kyoto Encyclopedia of Genes and Genomes (KEGG) enrichment analyses for significantly differentially abundant proteins (q-value < 0.05 and |log_2_FC| ≥ 1.0) [17]. Protein and protein interactions (PPIs) were predicted using STRING, and the resulting PPI network was visualized using Cytoscape 3.9.1. ChiPlot (https://www.chiplot.online/) (accessed on 15 June 2026) was used for plotting [18,19].

### 2.5. Drug Susceptibility Testing of V. alginolyticus to NOR in the Combination with Exogenous Metabolites

To evaluate the functional impact of nutritional inputs on norfloxacin (NOR) susceptibility, phenotypic growth curves were systematically monitored. Single bacterial colonies were inoculated into 5 mL of Luria–Bertani (LB) medium and cultured overnight at 30 °C with continuous agitation at 220 rpm. The resulting cultures were subsequently subcultured at a 1:100 dilution into fresh LB medium supplemented with NOR alone, individual exogenous metabolites alone, or their respective combinations. The baseline control group was maintained in raw LB medium without antibiotic or metabolic additives. All culture cohorts were incubated at 30 °C for 12 h, during which cellular proliferation was continuously quantified by measuring the optical density at 600 nm (OD600) utilizing an automated microplate reader (Bioscreen C, Oy Growth Curves Ab Ltd., Helsinki, Finland) [13]. A panel of 23 distinct exogenous metabolites was evaluated, with each compound tested at final working concentrations of 200, 400 and 800 μg/mL. The screened metabolic matrix comprised eleven amino acids (L-glycine, L-isoleucine, L-aspartate, L-leucine, L-proline, L-alanine, L-glutamine, L-arginine, L-tryptophan, L-asparagine, and L-serine), ten carbohydrates (D-glucose, maltose, trehalose, mannose, D-fructose, galactose, rhamnose, L-arabinose, raffinose, and melibiose), and two metabolic cofactors (vitamin B12 and folic acid). Every experimental condition was executed with a minimum of three independent biological replicates. To quantitatively evaluate growth inhibition, cumulative bacterial growth across 16 h was determined by calculating the area under the curve (AUC_0–16h_, OD_600_·h) via trapezoidal numerical integration. Statistical differences between the baseline NOR-alone control and metabolite co-treatment groups were analyzed using one-way analysis of variance (ANOVA) followed by Dunnett’s post hoc multiple comparisons test, with statistical significance defined at adjusted *p* < 0.05 (Supplementary File S1, Table S2; Supplementary File S2, Figure S2).

### 2.6. Quantification of Intracellular ROS Accumulation and Reduced Glutathione (GSH) Levels

Intracellular reactive oxygen species (ROS) generation was quantified utilizing the cell-permeable fluorogenic probe 2′,7′-dichlorodihydrofluorescein diacetate (DCFH-DA). Following diffusion across the bacterial cell membrane, the acetate groups are cleaved by intracellular esterases to yield non-fluorescent 2′,7′-dichlorodihydrofluorescein (DCFH), which is subsequently oxidized by intracellular ROS into the highly fluorescent compound 2′,7′-dichlorofluorescein (DCF). Following individual or combined exposures to norfloxacin (NOR) and exogenous metabolic supplements, cells were incubated with the probe according to standard loading protocols. The resulting DCF fluorescence intensity was recorded using a SpectraMax i3 multi-mode microplate reader (Molecular Devices, San Jose, CA, USA) with excitation and emission wavelengths configured at 495 and 525 nm, respectively [20]. Concurrently, intracellular reduced glutathione (GSH) concentrations were determined utilizing a commercial spectrophotometric assay kit (Nanjing Jiancheng Bioengineering Institute, Nanjing, China) in strict accordance with the manufacturer’s operational instructions. Absorbance profiles were recorded at 420 nm using the same SpectraMax i3 microplate platform. To ensure absolute experimental reproducibility and rigor, all physiological and biochemical assays were executed using three independent biological replicates, with each sample analyzed in technical triplicate.

### 2.7. RNA Extraction, Reverse Transcription, and Quantitative Real-Time PCR (qRT-PCR)

*Vibrio alginolyticus* cultures across all four experimental groups—untreated control, norfloxacin alone (NOR, 0.064 μg/mL, 1/2 MIC), glutamine alone (Gln, 400 μg/mL), and co-treatment (NOR + Gln)—were grown at 30 °C and harvested at the mid-exponential phase (OD600 = 1.0). Total RNA was extracted using the (Invitrogen, Carlsbad, CA, USA). RNA purity and concentration were assessed using a NanoDrop 2000 spectrophotometer (Thermo Fisher Scientific, Waltham, MA, USA) (A260/A280 ratio: 1.80–2.1), and RNA structural integrity was confirmed by 1.2% agarose gel electrophoresis. To thoroughly eliminate residual genomic DNA (gDNA) prior to reverse transcription, RNA samples were digested with a heat-labile double-strand-specific DNase (dsDNase; 37 °C for 2 min, followed by thermal inactivation at 65 °C for 2 min). Complementary DNA (cDNA) was synthesized from 1 μg of purified RNA using the PrimeScript RT reagent kit (Takara Bio Inc., Kusatsu, Japan).

Real-time PCR amplification was performed on a Bio-Rad CFX96 Real-Time PCR System using SYBR Green Master Mix. The thermal cycling conditions were: initial denaturation at 95 °C for 3 min, followed by 40 amplification cycles of denaturation at 95 °C for 10 s and annealing/extension at 60 °C for 30 s. Melting curve analysis was conducted immediately after cycling by continuously monitoring fluorescence from 65.0 °C to 95.0 °C at 0.5 °C increments. Kinetic reaction efficiencies (E = 80.1–91.2%, R^2^ > 0.998) were determined by four-parameter log-logistic curve fitting (l4 model) using the qpcR package in R Supplementary File S1 (Sheet: ‘Table S3’). Melting curve dissociation profiles confirmed sharp, single peaks without primer-dimers (<75.0 °C) across all assays Supplementary File S1 (Sheet: ‘Table S3’). The DNA gyrase subunit B (gyrB) gene was verified as a stably expressed internal reference (overall mean Ct = 21.69 ± 2.62, between-condition CV < 4.1%). Relative expression levels were calculated using the 2^−ΔΔCt^ method. Differences among biological triplicates were statistically analyzed by one-way ANOVA followed by Tukey’s honestly significant difference (HSD) post hoc test, with significance defined at *p* < 0.05. Detailed primer sequences and amplicon characteristics are listed in Supplementary File S1 (Sheet: ‘Table S3’).

## 3. Results

### 3.1. Growth Inhibition of V. alginolyticus by NOR

Growth-curve analysis was conducted to evaluate the response of *V. alginolyticus* to NOR at concentrations of 0.032, 0.064, and 0.128 μg/mL. As shown in Figure 1A, NOR inhibited bacterial growth in a concentration-dependent manner, with progressively stronger suppression observed at higher concentrations. The MIC of NOR against *V. alginolyticus* was determined to be 0.128 μg/mL via the broth microdilution assay (Supplementary File S2 (Supplementary Figure S3)). Among the tested concentrations, 0.064 μg/mL produced a clear inhibitory effect while maintaining measurable growth and was therefore selected for subsequent experiments.

### 3.2. Proteomic Profiling of V. alginolyticus Under NOR Treatment

To characterize proteomic alterations associated with NOR exposure, DIA-based quantitative proteomics was performed to compare untreated *V. alginolyticus* and NOR-treated *V. alginolyticus* (hereafter control and NOR, respectively). In total, 2693 proteins were confidently identified, meeting the criteria of a peptide-level FDR < 0.01 and at least two unique peptides per protein. High reproducibility among three independent biological replicates within each group was confirmed by Pearson correlation analysis (Figure 1B). Differential abundance analysis identified 580 differentially abundant proteins (DAPs) between control and NOR (Figure 1C); 412 proteins exhibited decreased abundance, whereas 168 proteins showed increased abundance under NOR treatment. Consistently, principal component analysis (PCA) showed a clear separation between control (*V. alginolyticus*) and NOR samples along principal component 1, indicating distinct global proteomic profiles following NOR treatment (Figure 1D).

### 3.3. Functional Enrichment Analysis of DAPs Under NOR Stress

To characterize the functional distribution of the DAPs, GO enrichment analysis was performed, covering the biological process, cellular component, and molecular function categories. In the biological process category, enriched terms included metabolic process, oxoacid metabolic process, and organic acid metabolic process, such as Generation of precursor metabolites and energy (FDR value = 2.02 × 10^−8^), Cellular respiration (1.9 × 10^−6^), Alpha-amino acid metabolic process (6.7 × 10^−6^), Cellular anatomical entity (1.63 × 10^−12^), Cytoplasm (5.1 × 10^−7^), Oxidoreductase activity (5.79 × 10^−9^), and Catalytic activity (3.15 × 10^−7^) etc. (Figure 2A). KEGG pathway enrichment analysis identified 12 significantly enriched pathways (*p* < 0.05), including microbial metabolism in diverse environments, the TCA cycle, carbon metabolism, and nitrogen metabolism, such as Metabolic pathways (2.17 × 10^−18^), Biosynthesis of secondary metabolites (3.76 × 10^−9^), Carbon metabolism (9.5 × 10^−9^), and Citrate cycle (TCA cycle) (9.31 × 10^−6^), etc. (Figure 2B). These enriched pathways were mainly associated with central metabolism.

### 3.4. PPI Network Analysis of DAPs in V. alginolyticus Under NOR Treatment

To elucidate the functional coordination and topological architecture of the differentially abundant proteins (DAPs) under norfloxacin (NOR) stress, a comprehensive protein–protein interaction (PPI) network was constructed utilizing the STRING database and visualized via Cytoscape. The global interaction network was filtered using the Molecular Complex Detection (MCODE) algorithm to identify highly interconnected functional sub-networks (clusters), revealing that a large proportion of downregulated proteins were tightly clustered within core metabolic pipelines (Figure 3). The network showed that many downregulated proteins were connected and mapped to central metabolic processes. Several metabolic enzymes, including SucD, SdhA, GcvP, Mdh_1, FrdA_2, GltA, SucA, SucC, FrdB and SdhB, showed reduced abundance under NOR treatment.

To identify the key topological nodes within the norfloxacin-responsive network, the Maximal Clique Centrality (MCC) algorithm in CytoHubba was applied to isolate the top 10 hub proteins within the DAPs (Figure 3F). These high-centrality hub proteins converged on the citrate cycle (TCA cycle), carbon metabolism, and cellular respiration. Topologically, SucA (Rank 1), Mdh_1 (Rank 2), and GltA (Rank 3) emerged as the highest-scoring nodes, closely supported by SucC, FrdA_2, FrdB, SdhA, SucD, and SdhB, with GcvP at position 10 (Figure 3F). The coordinated downregulation of these high-centrality hub proteins reflects an extensive topological downshift across the core metabolic architecture.

### 3.5. Effects of Exogenous Metabolites on NOR-Treated V. alginolyticus

Based on the pathway enrichment in the proteomic dataset, whether exogenous metabolites modulate the growth phenotype of *V. alginolyticus* under NOR exposure was assessed utilizing OD600-based growth curves. A total of 23 metabolites were used, including glucose (involved in EMP, PPP and TCA cycle); maltose, trehalose, mannose, fructose, galactose and rhamnose (carbohydrate metabolism); arabinose (PPP); raffinose and melibiose (EMP); vitamin B12 (coenzyme in fatty acid degradation); glycine, leucine, alanine, arginine, aspartate, asparagine, isoleucine, proline, tryptophan, glutamine, serine (amino acid metabolism); and folic acid (metabolism of cofactors and vitamins). To determine the effects of these metabolites on *V. alginolyticus*, the growth curve was measured in LB medium with 0.064 μg/mL NOR added at each of the three concentrations, or without adding NOR. Compared with the treatment group that only added metabolites, 11 metabolites (shown in Figure 4A–K) showed a significant decrease in growth in the LB medium containing NOR: glycine, isoleucine, vitamin B12, aspartate, leucine, proline, alanine, glutamine, arginine, tryptophan, and glucose. Consistent with the OD_600_ curves in Figure 4A–K, these metabolites had little to modest effects on growth in LB alone, but in the presence of NOR they further reduced OD_600_ relative to NOR alone, indicating enhanced NOR-mediated growth inhibition, often in a dose-dependent manner. To quantitatively evaluate the synergistic antibacterial efficacy, cumulative bacterial growth across 16 h was integrated using the area under the curve (${\mathrm{AUC}}_{0–16h}$), and the corresponding AUC comparison charts for these 11 candidate metabolites in combination with NOR are provided in the Supplementary Material (Supplementary File S1 (Sheet: ‘Table S2’) and Supplementary File S2 (Supplementary Figures S1 and S2)). In addition, the remaining 12 metabolites did not change compared with the treatment group: folic acid, serine, asparagine, maltose, trehalose, fructose, galactose, mannose, raffinose, melibiose, rhamnose, arabinose (shown in Figure S1). These results indicate that NOR may affect carbon metabolism and energy metabolism aspects.

### 3.6. Core Metabolic Pathway Remodeling and Norfloxacin Susceptibility Dynamics

As summarized in Figure 5, NOR treatment was associated with coordinated downregulation of proteins mapped to central carbon and energy metabolism, including the EMP pathway, the PPP, and the TCA cycle, together with broad remodeling of amino acid metabolism. Consistent with the pathway map (Figure 5), these changes converge on reduced metabolic flux into pyruvate/acetyl-CoA and key TCA hubs (oxaloacetate and 2-oxoglutarate), with downstream implications for NADH supply and oxidative phosphorylation. The amino acid changes involved multiple interconnected modules, including the glutamine-glutamate axis, the arginine-proline branch, the aspartate family, and pathways linked to aromatic and branched-chain amino acid metabolism. In contrast, a limited set of energy-related proteins increased in abundance, suggesting that selected components of respiratory capacity may be retained while central carbon supply is constrained. Consistent with this network-level view, phenotypic co-treatment screening showed selective metabolite-enabled potentiation of NOR-mediated growth inhibition, with glucose, glycine, tryptophan, and the amino acids glutamine, proline, arginine, isoleucine, and aspartate showing measurable potentiating effects. Together, Figure 5 provides an integrated framework that links proteomic directionality to entry-point-dependent carbon and nitrogen inputs feeding the TCA cycle and oxidative phosphorylation as examined in detail in the pathway-specific sections below.

### 3.7. Target Node Verification: Exogenous Metabolites Alter Intracellular ROS/GSH Balance and Relieve Transcriptional Suppression Under Norfloxacin Challenge

To validate the functional impact of the identified metabolic nodes, physiological shifts and mRNA expression changes were evaluated under NOR challenge (Figure 6). As shown in Figure 6A,B, NOR monotherapy (0.064 μg/mL) induced an increase in ROS fluorescence intensity accompanied by a reciprocal reduction in intracellular GSH levels compared to the untreated control group. Under baseline conditions without antibiotic stress, glycine supplementation alone did not alter intracellular ROS levels compared to the control (*p* > 0.05, letter ‘d’; Figure 6A), while resulting in a slight increase in basal reduced GSH content (*p* < 0.05, letter ‘a’ vs. ‘b’; Figure 6B). However, the co-administration of exogenous glycine (200 and 400 μg/mL) with NOR markedly intensified oxidative stress, resulting in a synergistic, dose-dependent escalation of DCF fluorescence alongside a severe depletion of the intracellular reduced GSH pool below the NOR-alone baseline (Figure 6A,B).

For the parallel glutamine/glutamate-to-2-oxoglutarate axis, qPCR was performed to monitor four target genes downstream of glutamine catabolism: NAD-glutamate dehydrogenase (N646_0629) and three components of the 2-oxoglutarate dehydrogenase complex (N646_1848, N646_2999, and N646_3000). mRNA expression analysis revealed that individual NOR exposure systematically downregulated all four transcripts compared to the untreated control group (Figure 6C). Although individual Gln supplementation resulted in transcript levels lower than the untreated control, the co-administration of Gln and NOR effectively alleviated NOR-induced transcriptional repression, significantly restoring the expression of these central metabolic genes relative to the NOR-alone group (Figure 6C).

## 4. Discussion

Antibiotic tolerance can evolve rapidly under repeated antibiotic exposure and can accelerate the emergence of resistance [21]. Because antibiotic efficacy is tightly linked to bacterial metabolic state [22], defining the metabolic response to NOR can help explain tolerance-like phenotypes and identify metabolite-based potentiation strategies in *V. alginolyticus*. In line with this rationale, quantitative proteomic analysis revealed that NOR exposure induces extensive proteome remodeling in *V. alginolyticus*, with significant downregulation in the abundance of proteins involved in central metabolic pathways. In this study, several DAPs associated with the generation of precursor metabolites and energy were significantly downregulated under NOR exposure, including mdh (Malate dehydrogenase), sucC (Succinate–CoA ligase [ADP-forming] subunit beta), sucD (Succinate–CoA ligase [ADP-forming] subunit alpha), glgA (Glycogen synthase), glgC (Glucose-1-phosphate adenylyltransferase), CcoO (Cytochrome c oxidase, subunit), and napA (Periplasmic nitrate reductase). The down-regulation of these proteins indicates that *V. alginolyticus* decreases the expression of core bioenergetic, carbohydrate storage, and respiratory machinery to maximize metabolic efficiency and resource conservation under NOR-induced bactericidal stress, which is a conserved adaptive response of bacteria to antibiotic-mediated challenges. Enrichment and network analyses converged on the EMP, PPP, the TCA cycle, together with connected amino acid pathways, indicating coordinated downregulation of central carbon and energy metabolism-related proteins under NOR stress (Figure 5). Fluoroquinolones generate cytotoxic DNA lesions by trapping DNA gyrase and topoisomerase IV cleavage complexes, yet antibacterial outcomes depend strongly on physiological state and stress responses rather than target engagement alone [23]. This pattern is consistent with tolerance and persistence frameworks, in which reduced metabolic flux and altered energy and redox coupling support survival under bactericidal stress without requiring genetic resistance [5,24,25]. Together, these results support the interpretation that NOR is associated with a low-flux physiological state in *V. alginolyticus*, consistent with norfloxacin-associated remodeling of metabolic proteins reported in a quantitative proteomics study of *A. hydrophila* [13].

Only a narrow subset of metabolites potentiated NOR-mediated growth inhibition, whereas most supplements showed no obvious effect (Figure 4). This selectivity suggests that potentiation depends on specific metabolic entry points rather than a general nutrient effect [26,27,28]. Related metabolite-enabled sensitization has also been reported in other pathogens, including amino-acid metabolism modules that potentiate aminoglycoside killing or weaken stress-associated resistance [29,30]. Because respiration and cellular energetics modulate antibiotic efficacy and are often perturbed during bactericidal stress [10,31,32,33], effective potentiators may restore respiratory engagement and redox/energy balance, thereby increasing susceptibility under NOR.

To bridge the proteomic directionality with phenotypic outcomes, node-specific validation workflows focusing on the perturbation of cellular redox homeostasis were executed. Physiological tracking revealed that while glycine monotherapy remained metabolically neutral, its co-administration with NOR drove a synergistic escalation of intracellular ROS alongside a severe depletion of the intracellular GSH pool. Our biochemical measurements demonstrate that norfloxacin–glycine co-treatment is accompanied by elevated ROS-associated fluorescence and a concomitant decrease in measurable intracellular reduced GSH content. We hypothesize that enhanced ROS accumulation under co-treatment may facilitate the rapid oxidation of reduced GSH to GSSG, thereby taxing endogenous redox buffering capacity. However, because specific enzyme activities within the glutathione metabolic pathway (such as glutathione reductase, glutathione peroxidase, or γ-glutamylcysteine synthetase) and direct metabolic flux were not measured in this study, whether this GSH reduction stems from accelerated turnover, attenuated biosynthesis, or impaired recycling remains to be functionally verified. Further targeted enzymological assays and fluxomic profiling will be essential to delineate the precise enzymatic determinants underlying this redox perturbation.

In the second validation workflow, the parallel Gln/glutamate-to-2-oxoglutarate entry axis was interrogated via qPCR to track the transcriptional plasticity of five core bottleneck enzymes. These targets comprised NAD-glutamate dehydrogenase (N646_0629), three components of the 2-oxoglutarate dehydrogenase complex (N646_1848, N646_2999, and N646_3000). Transcriptional analysis showed that while individual NOR exposure led to a severe downregulation of these transcripts, the supplementary addition of Gln significantly reversed this genetic suppression. Collectively, these complementary lines of evidence suggest that targeted anaplerotic inputs can effectively attenuate norfloxacin insensitivity. By exploiting entry-point-specific vulnerabilities, these exogenous metabolites override defensive metabolic constraints and prompt transcriptional reactivation of the suppressed TCA cycle architecture, offering a testable framework for metabolite-enabled antibiotic potentiation.

Within the EMP pathway, phosphoglycerate kinase was downregulated, consistent with reduced glycolytic capacity and potentially reduced pyruvate-derived acetyl-CoA supply. Glucose potentiation was transient, supporting a state-dependent model in which NOR-treated cells are constrained by downstream metabolic and/or redox limitations [22,31,34]. This interpretation could be tested using time-resolved measurements of oxygen consumption, membrane potential, and the NADH/NAD^+^ ratio [35].

PPP-associated proteins showed a selective shift under NOR. Zwf and Pgl were downregulated, whereas Rpe and Tal were upregulated (Figure 5). This pattern is consistent with PPP remodeling that could alter NADPH supply relevant to redox buffering during fluoroquinolone stress [18,31,36]. Because proteomics cannot distinguish redox limitation from precursor limitation, targeted metabolomics and isotope tracing focused on the NADPH/NADP^+^ ratio and PPP metabolic flux and nucleotide-pool changes would be informative, particularly in potentiator versus non-potentiator conditions.

Only a narrow subset of amino acids potentiated NOR-associated growth inhibition, including glutamine, proline, arginine, isoleucine, and aspartate (Figure 4 and Figure 5). Notably, these effective inputs map to TCA-linked hub entry points connected to 2-oxoglutarate and oxaloacetate, supporting an entry-point-specific potentiation effect rather than a general nutrient effect [27,28]. This convergence also aligns with respiration and bioenergetic models that link antibiotic outcomes to energetic set points and redox coupling [24,31]. A bounded working model is that potentiating amino acids relieve anaplerotic constraints and shift cellular energy/redox balance, increasing the likelihood that NOR-associated lesions progress to sustained growth inhibition. Consistent with this model, potentiators would be expected to preferentially alter TCA-hub intermediates (2-oxoglutarate and oxaloacetate) and NADH/NAD^+^ and/or NADPH/NADP^+^ balance relative to non-potentiators under NOR.

Although central metabolism was broadly suppressed, a limited set of oxidative phosphorylation-related proteins increased in abundance, including ATP synthase subunits AtpE and AtpB, suggesting partial retention of respiratory machinery despite constraints in substrate and reducing equivalent supply (Figure 5). Links between respiration, bioenergetics, and antibiotic efficacy have been reported across bacteria, and bactericidal treatment is frequently accompanied by characteristic metabolic and redox perturbations [31,37]. In this context, redox-active metabolites have been shown to remodel tolerance-associated bioenergetic states and thereby alter antibiotic outcomes [24]. Moreover, metabolite stimulation can sensitize tolerant cells to fluoroquinolones by shifting electron transport metabolic flux and redox balance [22]. Together with the selective potentiation phenotype observed here (Figure 4 and Figure 5), these observations support a conditional model in which NOR outcomes depend on shifts in respiratory metabolic flux and redox balance [22,31]. Given this context, ROS is best treated as a testable mechanism rather than an inference, given evidence for ROS-independent killing and methodological limitations [38,39]. Oxygen-dependence assays and orthogonal ROS readouts can determine whether ROS contributes to potentiation under NOR co-treatment [40,41].

A vitamin B12-associated node was upregulated under NOR, including N646_2085 (Figure 5). This suggests that cobalamin-linked routing may remain accessible, but this inference is hypothesis-generating because abundance changes do not establish metabolic flux directionality [42]. Testing vitamin B12 supplementation under NOR co-treatment, or genetically perturbing the corresponding B12-dependent enzyme, could determine whether this node contributes to potentiation.

Overall, NOR exposure was associated with coordinated downregulation of proteins spanning the EMP, PPP, the TCA cycle, and amino acid metabolism, consistent with a low-flux adaptive state [19,20,21]. Only a defined subset of metabolites potentiated NOR-associated growth inhibition, supporting metabolite-specific potentiation tied to metabolic and redox remodeling rather than a generic nutrient effect [22,31]. Figure 5 therefore serves as an integrated overview linking proteomic directionality with metabolite phenotypes, not a reconstructed metabolic network. Because protein abundance does not directly report metabolic flux or intracellular drug levels, these results are interpreted as a testable mechanistic framework capable of guiding targeted validation [22,31].

## 5. Conclusions

In this study, we investigated the adaptive physiological responses of *Vibrio alginolyticus* to sub-inhibitory NOR stress. Our empirical findings demonstrate that NOR exposure induces a marked deceleration of bacterial growth, accompanied by a quantitative proteomic downshift in central carbon metabolism and the TCA cycle. Through phenotypic screening, we identified that supplementation with specific exogenous metabolites, notably glycine and glutamine, effectively potentiates NOR-induced growth inhibition. Furthermore, direct biochemical assays confirmed that this metabolite–antibiotic co-treatment triggers a significant accumulation of intracellular reactive oxygen species (ROS) alongside a severe depletion of the intracellular reduced GSH pool.

Drawing upon these direct observations, we propose a conceptual mechanistic model for this phenotypic potentiation. We hypothesize that exogenous glycine and glutamine act as metabolic drivers that circumvent the NOR-induced low-flux state, reactivating the TCA cycle and cellular respiration. This forced metabolic stimulation may contribute to the elevated ROS accumulation. We hypothesize that this ROS accumulation overwhelms the endogenous antioxidant capacity—manifesting as the observed GSH depletion—and ultimately disrupts cellular redox homeostasis. While future fluxomic and time-kill validations are required to confirm real-time metabolic kinetics and bactericidal causality, this conceptual framework highlights the potential of metabolic reprogramming as a viable strategy to resensitize aquaculture pathogens to existing fluoroquinolones.

## Figures and Tables

**Figure 1:**
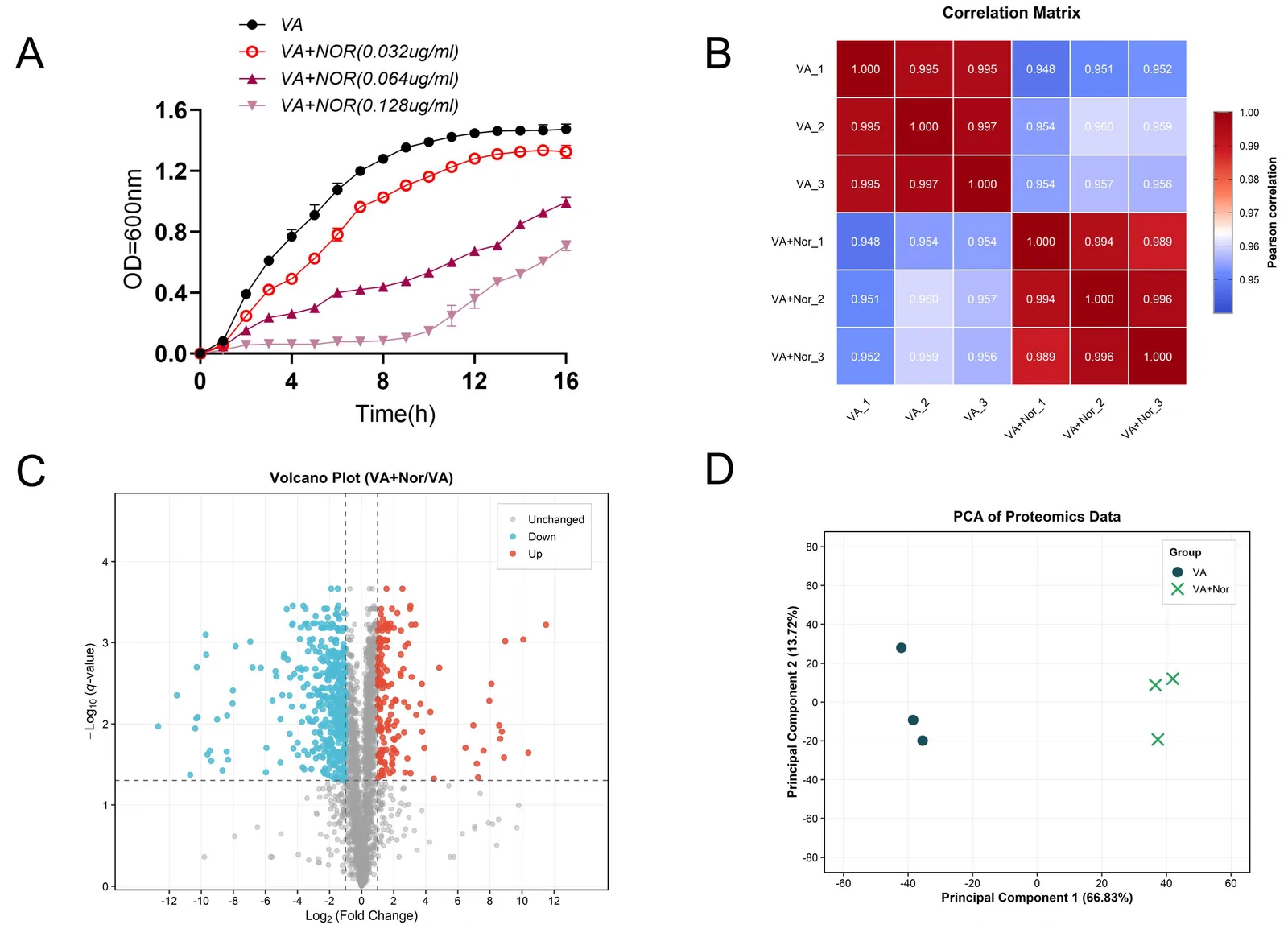
Quantitative proteomics overview of *V. alginolyticus* under NOR treatment. (**A**) Growth curves of *V. alginolyticus* under different NOR concentrations. Data are presented as mean ± SD (n = 3). (**B**) Heat map of Pearson correlation coefficients among three biological replicates within each group. (**C**) Volcano plot showing protein abundance differences between control and NOR; proteins with decreased abundance are indicated in blue, whereas proteins with increased abundance are indicated in red (log_2_(Fold Change) > 1, q-value < 0.05), downregulated proteins in blue (log2(Fold Change) > −1, q-value < 0.05), and non-significant proteins in grey. The vertical dashed lines indicate the fold-change thresholds. (**D**) Principal component analysis (PCA) of the proteomics data, indicating separation between control and NOR.

**Figure 2:**
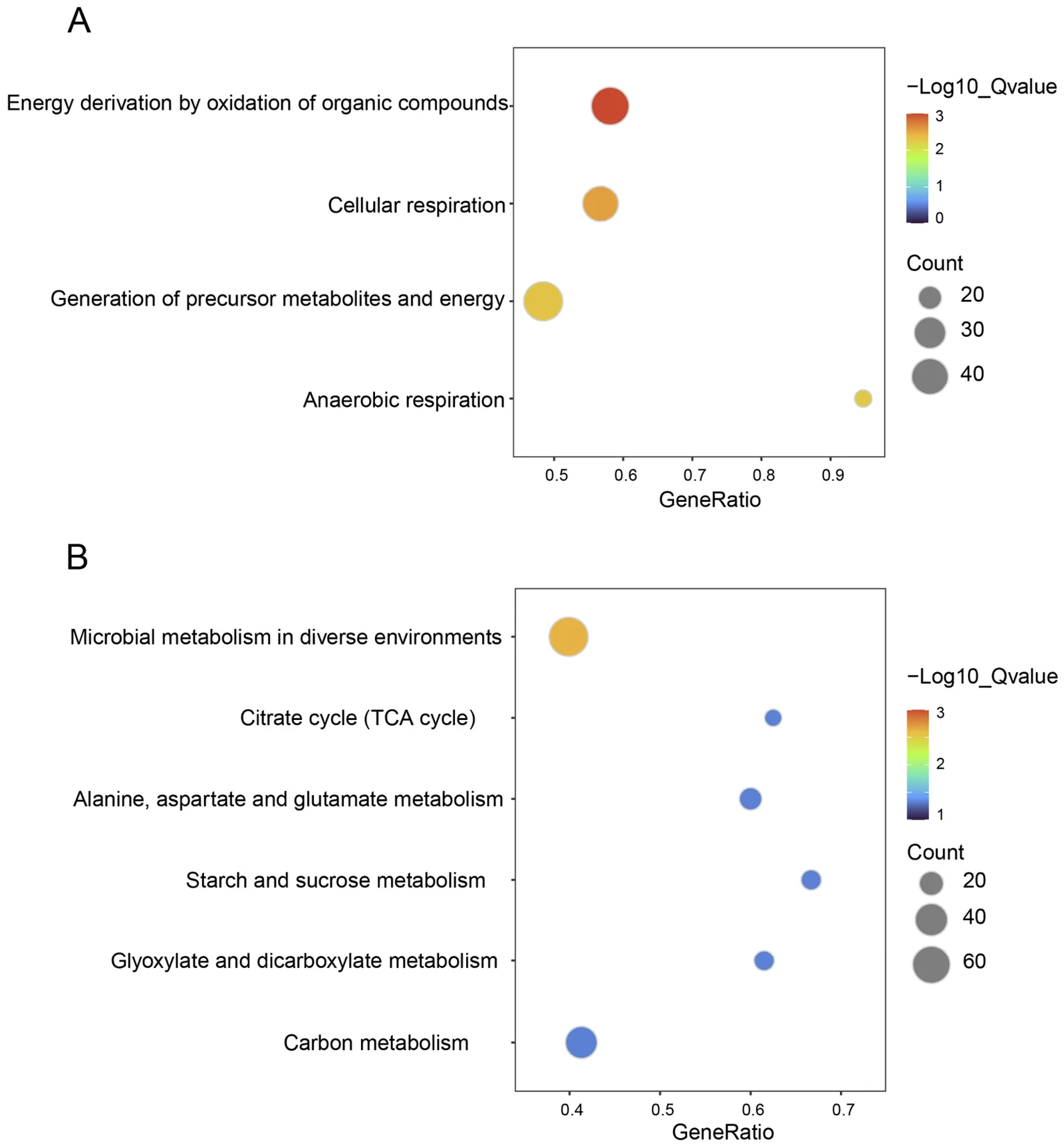
Functional enrichment overview of differentially abundant proteins (DAPs) in *Vibrio alginolyticus* under norfloxacin (NOR) treatment. (**A**) Bubble plot of significantly enriched Gene Ontology (GO) biological process (BP) terms (including energy derivation by oxidation of organic compounds, cellular respiration, generation of precursor metabolites and energy, and anaerobic respiration). (**B**) Bubble plot of enriched Kyoto Encyclopedia of Genes and Genomes (KEGG) pathways. In both panels, the *x*-axis indicates the gene ratio (GeneRatio), and bubble size corresponds to the number of identified target proteins (Count). The color gradient from blue through yellow to red represents statistical significance based on Benjamini–Hochberg adjusted false discovery rates (−log_10_(*p*-value)).

**Figure 3:**
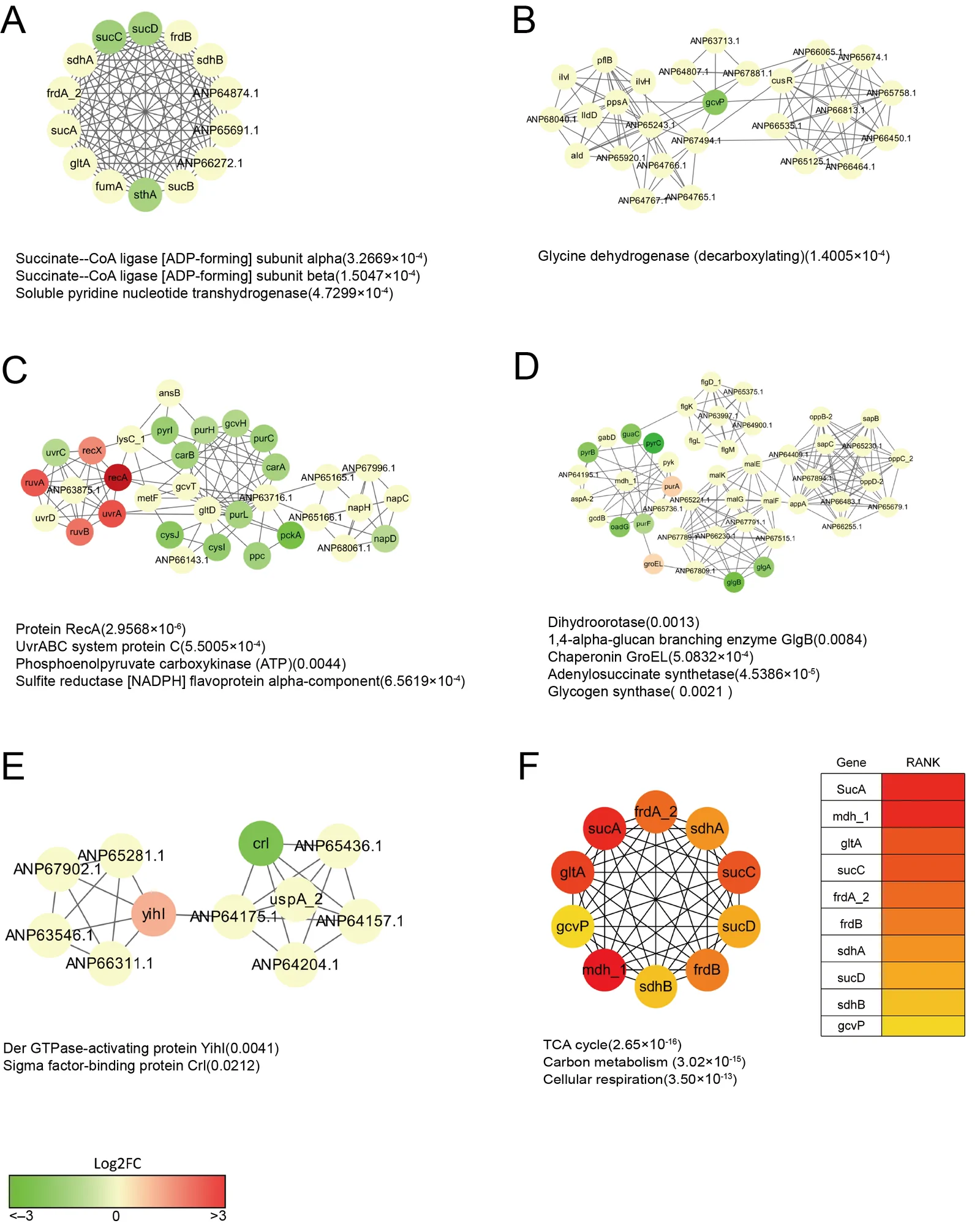
Protein–protein interaction (PPI) network and topological cluster analysis of differentially abundant proteins (DAPs) under norfloxacin (NOR) treatment. (**A**–**E**) Highly interconnected sub-networks (clusters) extracted from the global DAP interaction network using the MCODE algorithm. Enriched functional annotations and representative functional enzymes (with associated significance values) are highlighted below each panel. Node colors represent log_2_(Fold Change) values under NOR stress, transitioning from green (downregulated, ≤−3.0) through pale yellow (0) to red (upregulated, ≥3.0). (**F**) Sub-network of the top 10 hub proteins identified via the maximal clique centrality (MCC) algorithm in CytoHubba, which predominantly converge on the citrate cycle (TCA cycle), carbon metabolism, and cellular respiration. In the network and corresponding rank table, node color reflects topological ranking (MCC score), with red indicating highest centrality (Rank 1: SucA) descending to yellow (Rank 10: gcvP).

**Figure 4:**
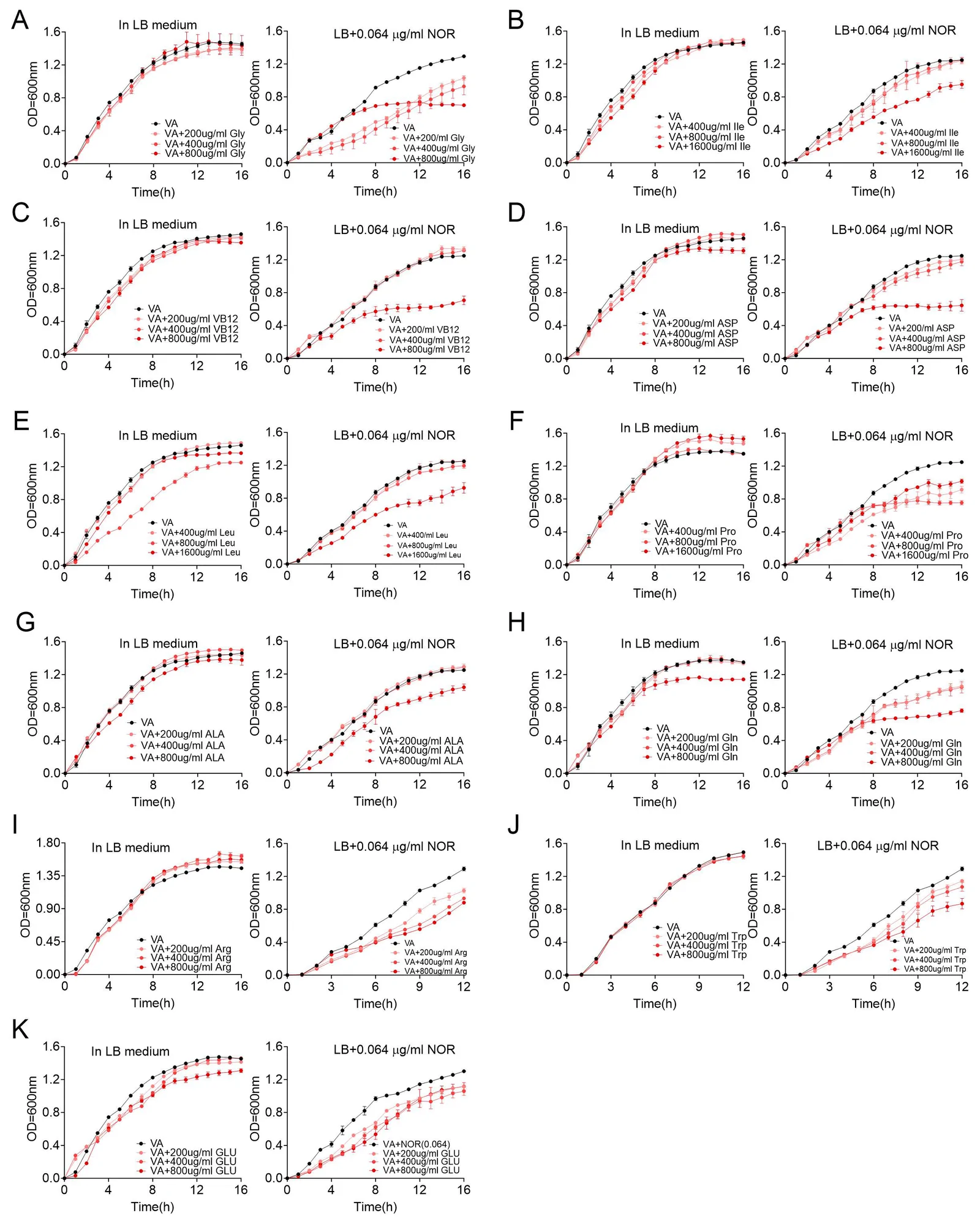
Growth curves of *V. alginolyticus* in LB medium with or without 0.064 μg/mL NOR supplemented with serial doses of exogenous metabolites. Panels (**A**–**K**) show cultures supplemented with glycine, isoleucine, vitamin B12, aspartate, leucine, proline, alanine, glutamine, arginine, tryptophan, and glucose, respectively, at 200, 400, and 800 μg/mL. LB medium with and without 0.064 μg/mL NOR served as controls.Data are presented as mean ± SD (*n* = 3)

**Figure 5:**
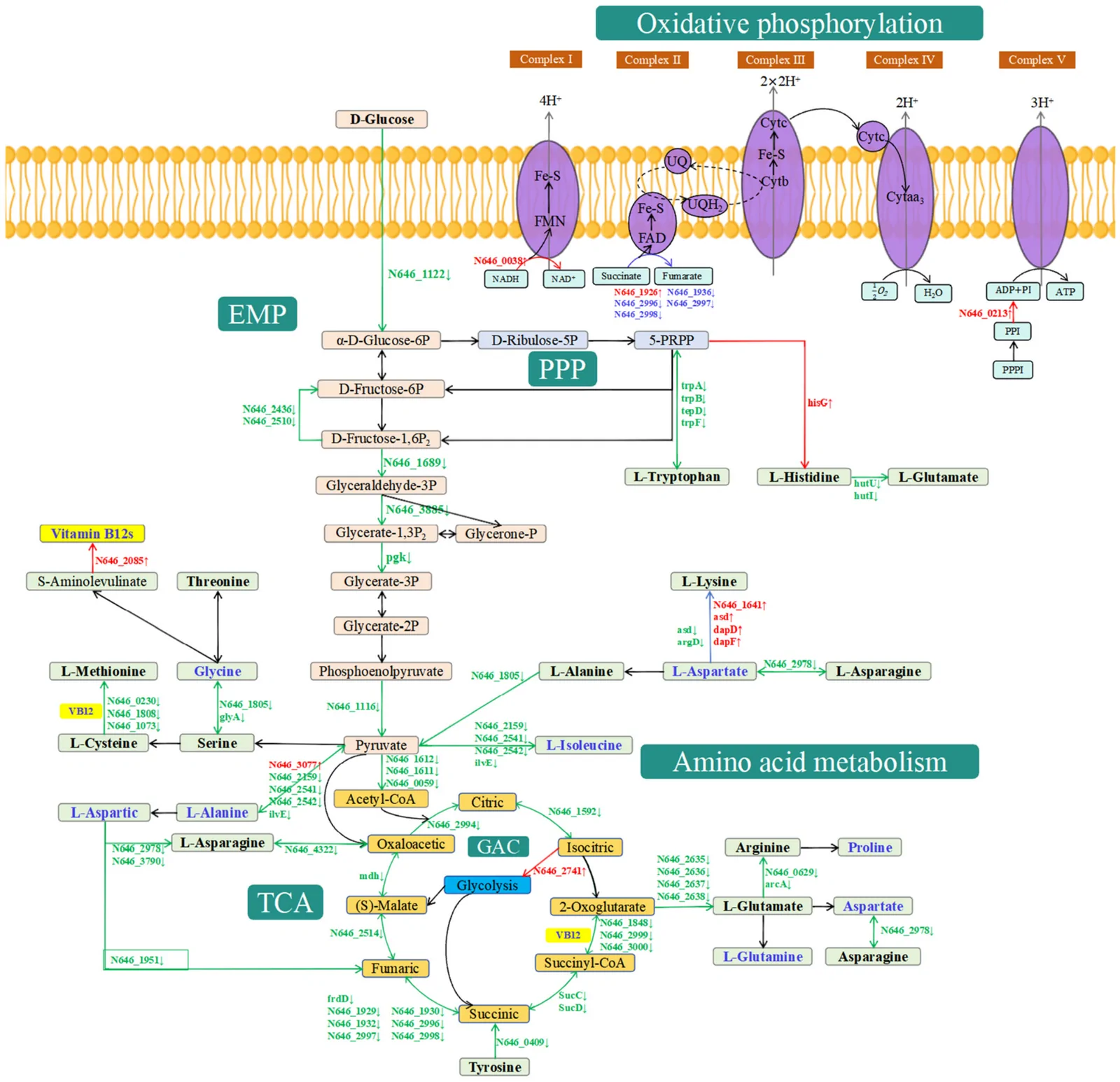
Integrated schematic diagram illustrating metabolic remodeling and redox perturbations in Vibrio alginolyticus under norfloxacin (NOR) challenge. Central metabolic pathways—including glycolysis (EMP, Embden–Meyerhof–Parnas pathway), the pentose phosphate pathway (PPP), the tricarboxylic acid (TCA) cycle, the glyoxylate cycle (GAC), amino acid metabolism, and electron transport chain/oxidative phosphorylation complexes (Complexes I–V)—are comprehensively integrated. Differentially abundant proteins (enzymes) identified by quantitative proteomics are designated by gene names or locus tags, with green and red text indicating downregulated and upregulated proteins/enzymes under NOR stress, respectively. Green and red arrows indicate metabolic steps catalyzed by downregulated and upregulated enzymes, respectively. Candidate exogenous metabolites and pathway intermediates are distinguished by different boxes: blue-shaded boxes indicate exogenous metabolites that significantly augmented NOR-induced growth inhibition (synergistic antimicrobial potentiation); yellow-shaded boxes denote core TCA cycle intermediates and vitamin B12 precursors; and plain black-bordered boxes indicate other pathway intermediates or non-sensitizing metabolites. Arrows indicate metabolic fluxes: black solid and dashed arrows denote direct biochemical transitions and multi-step or electron transfer cascades, respectively

**Figure 6:**
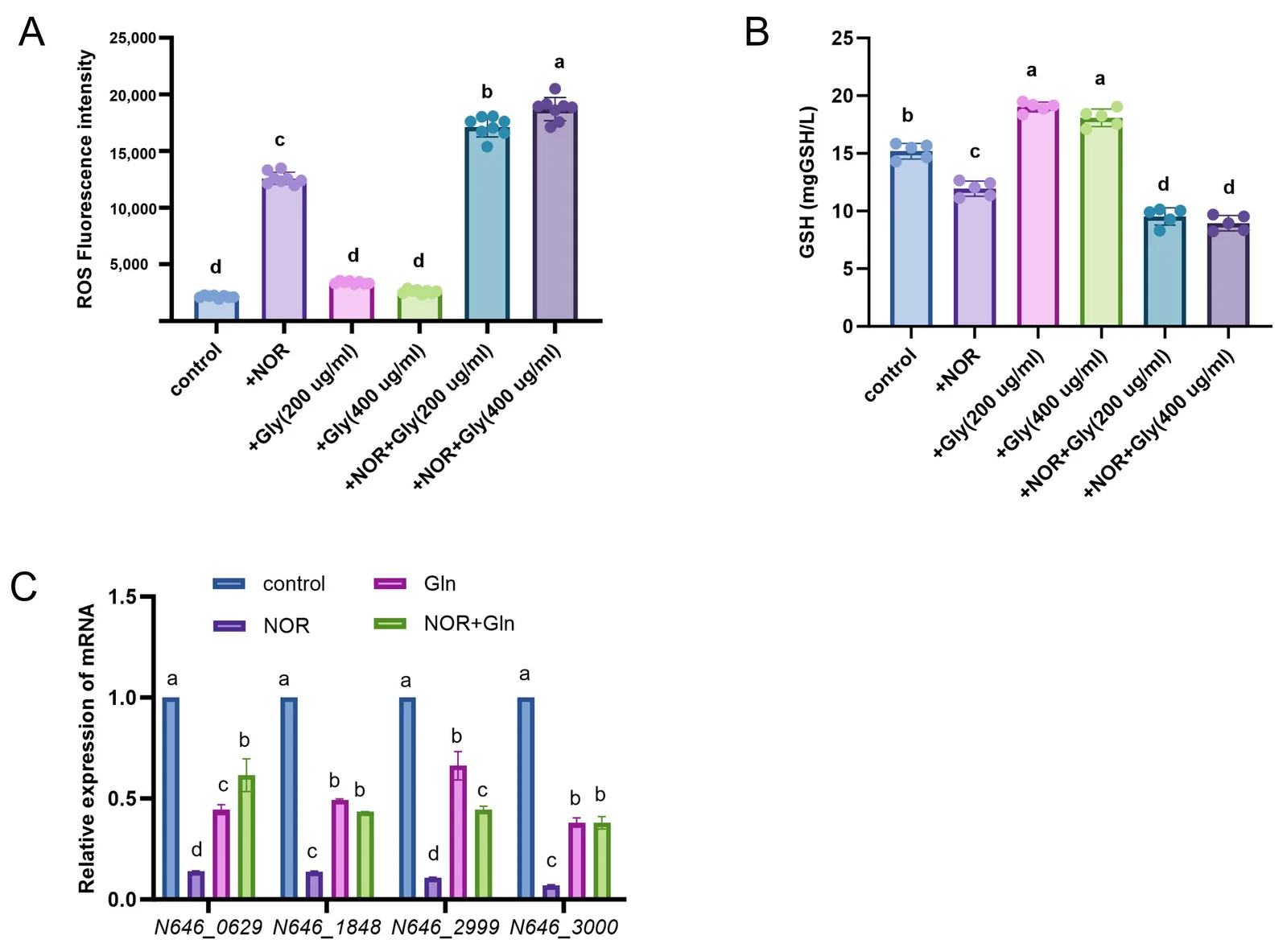
Physiological stress profiles and transcriptional plasticity of *Vibrio alginolyticus* under individual and combined antibiotic-metabolite treatments. (**A**) Intracellular reactive oxygen species (ROS) levels measured by DCFH-DA fluorescence intensity (n = 3) and (**B**) intracellular reduced glutathione (GSH) concentrations (mg GSH/L) under norfloxacin (NOR, 0.064 μg/mL) stress supplemented with or without exogenous glycine (Gly, 200 and 400 μg/mL) (n = 3). (**C**) Relative mRNA expression levels of four core anaplerotic bottleneck transcripts (N646_0629, N646_1848, N646_2999, and N646_3000) normalized to gyrB following exposure to NOR (0.064 μg/mL), exogenous glutamine (Gln, 400 μg/mL), or their combination (NOR + Gln). Bars represent the mean ± standard deviation (SD) (n = 3). Distinct lowercase letters above the bars indicate statistically significant differences (*p* < 0.05) determined by one-way ANOVA followed by Tukey’s post hoc pairwise multiple comparisons.

## Data Availability

Raw mass spectrometry proteomics data for peptides have been deposited in the ProteomeXchange Consortium via the iProX partner repository with the dataset identifier PXD072925.

## Supplementary Materials

The following supporting information can be downloaded at: https://www.mdpi.com/article/10.3390/life16101607/s1.
