# Supplementary material for: Quantitative Proteomic Analysis Reveals That Central Metabolic Pathways Influence Norfloxacin Susceptibility in Vibrio alginolyticus
Source: Life (Basel). 2026 Sep 25;16(10):1607. doi: 10.3390/life16101607 (PMC13705885; doi:10.3390/life16101607)
Supplement: Supplementary file 1 [file life-16-01607-s001.zip › Supplementary File S2.pdf]

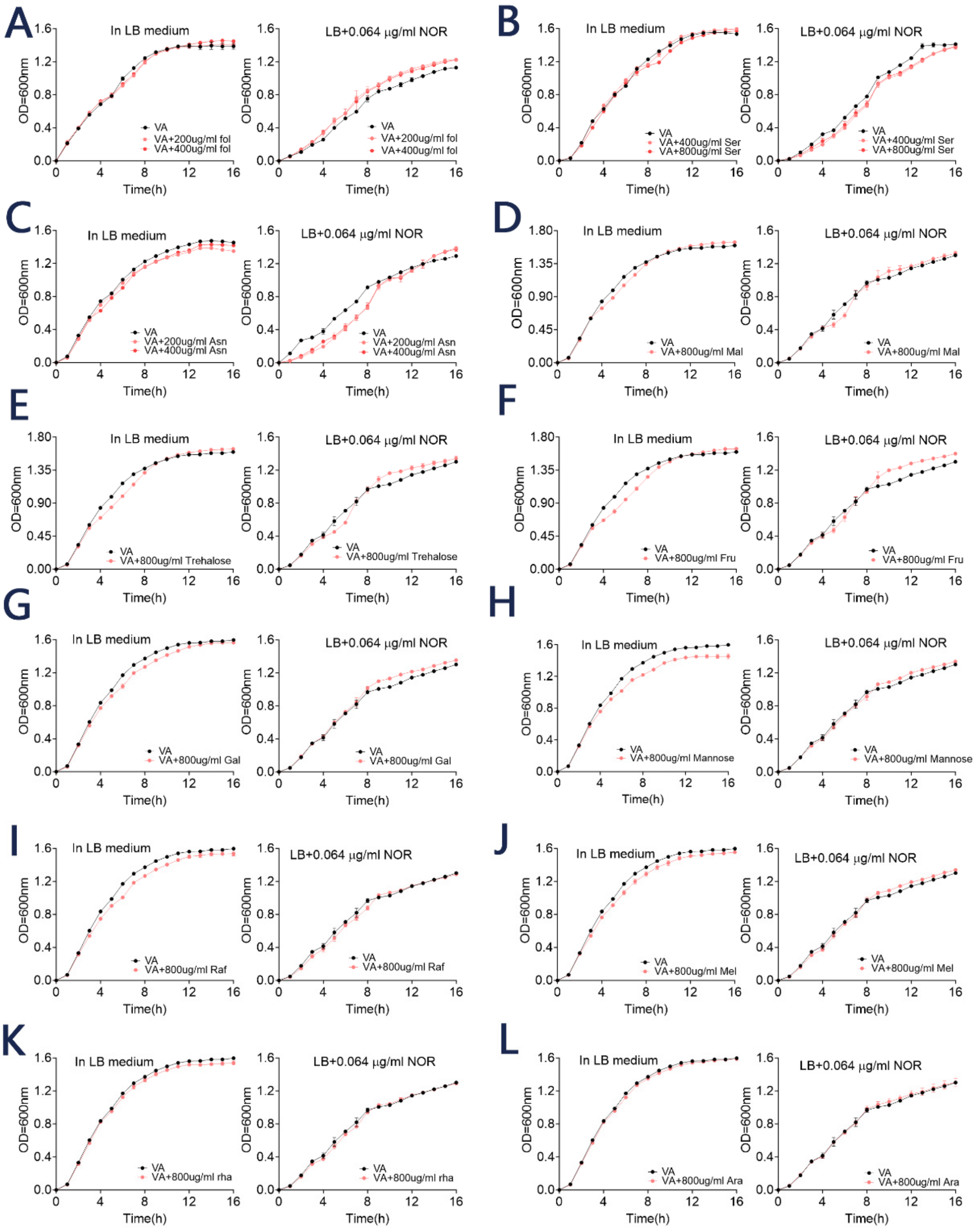

Figure S1. Growth curves of *V. alginolyticus* in LB medium with or without 0.064 μg/mL NOR supplemented with serial doses of exogenous metabolites. Panels A-L show culture supplemented with folic acid and asparagine at 200 and 400 μg/mL， serine at 400 and 800 μg/mL, and maltose, trehalose, fructose, galactose, mannose, raffinose, melibiose, rhamnose ， and arabinose at 800 μg/mL. LB medium with and without 0.064 μg/mL NOR served as controls.

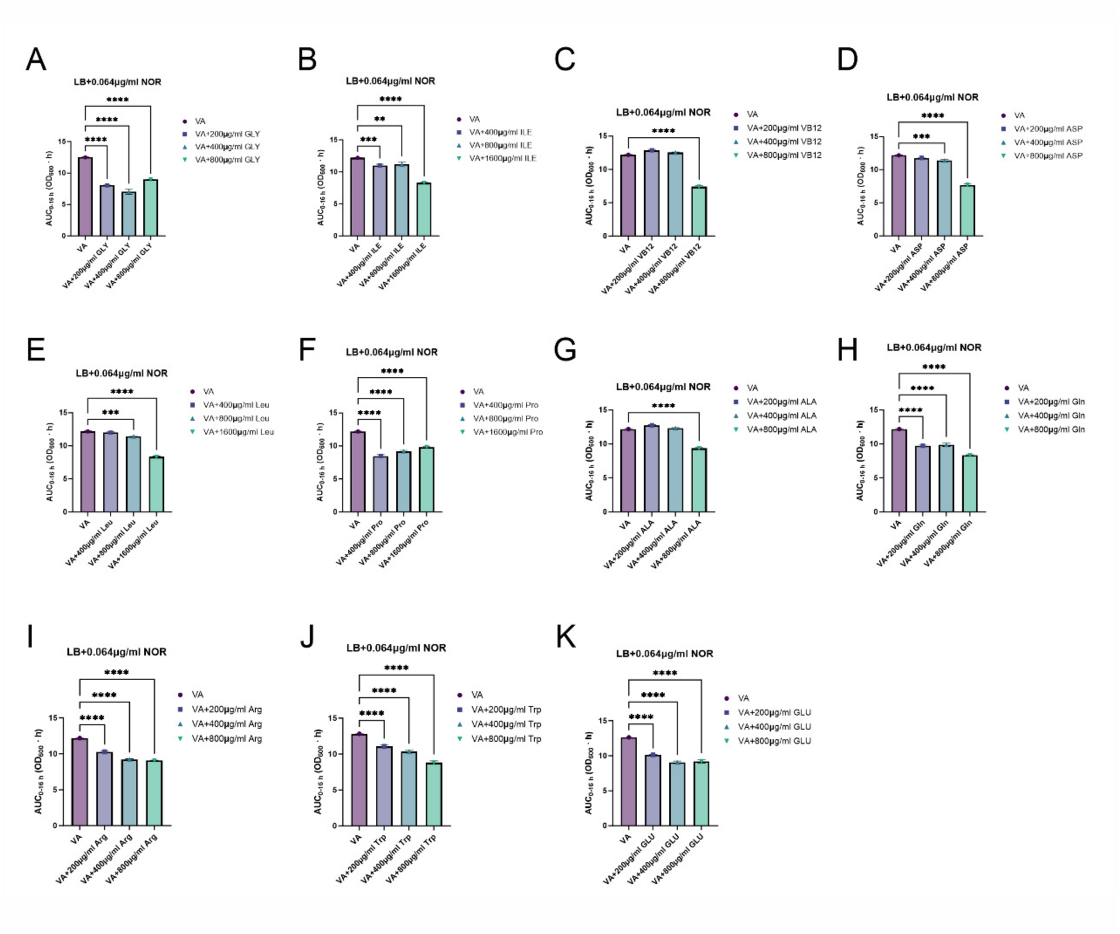

Figure S2. Quantitative assessment of Vibrio alginolyticus growth inhibition by norfloxacin (NOR) in combination with candidate exogenous metabolites based on area under the curve (AUC).

(A–K) Bar charts displaying the $AUC_{0\text{-}16\ h}$ (OD600·h) calculated from 16-h growth curves of *V. alginolyticus* cultured in LB medium containing 0.064 μg/mL NOR (baseline control, designated as VA) and co-supplemented with three graded concentrations of individual metabolites: (A) glycine (GLY; 200, 400, and 800 μg/mL), (B) L-isoleucine (ILE; 400, 800, and 1600 μg/mL), (C) vitamin B12 (VB12; 200, 400, and 800 μg/mL), (D) L-aspartate (ASP; 200, 400, and 800 μg/mL), (E) L-leucine (Leu; 400, 800, and 1600 μg/mL), (F) L-proline (Pro; 400, 800, and 1600 μg/mL), (G) L-alanine (ALA; 200, 400, and 800 μg/mL), (H) L-glutamine (Gln; 200, 400, and 800 μg/mL), (I) L-arginine (Arg; 200, 400, and 800 μg/mL), (J) L-tryptophan (Trp; 200, 400, and 800 μg/mL), and (K) D-glucose (GLU; 200, 400, and 800 μg/mL). Bar heights represent the mean ± SD (n = 3 independent biological replicates), with individual data points overlaid. Statistical differences between the baseline NOR group and each co-treatment condition were determined by one-way ANOVA followed by Dunnett's multiple comparisons test (residual df = 8). Asterisks denote statistically significant reductions in growth relative to the NOR-alone control ($P < 0.05$, ** $P < 0.01$, *** $P < 0.001$, **** $P < 0.0001$; ns, not significant). Comprehensive numerical

data—including exact mean AUC values, ΔAUC, 95% confidence intervals, inhibition percentages, Dunnett's q statistics

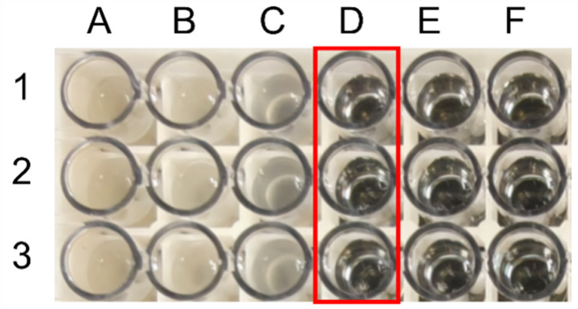

Figure S3. Determination of the minimum inhibitory concentration (MIC) of norfloxacin (NOR) against Vibrio alginolyticus. Broth microdilution assay was performed in a 96-well microtiter plate using two-fold serial dilutions of norfloxacin across columns A–F (0.016, 0.032, 0.064, 0.128, 0.256, and 0.512 μg/mL, respectively). Rows 1–3 represent three independent technical replicates. Wells with visible bacterial turbidity indicate bacterial growth (columns A–C), whereas clear wells indicate complete growth inhibition (columns D–F). The MIC of NOR against V. alginolyticus was determined to be 0.128 μg /mL (column D, highlighted by the red box), defined as the lowest antibiotic concentration that completely inhibited visible bacterial growth. Consequently, 0.064 μg /mL (column C, corresponding to 1/2 MIC) was selected as the sub-inhibitory concentration for subsequent physiological and proteomic investigations.
